# Analysis of the Static Behavior of a Single Tree on a Finite Element Model

**DOI:** 10.3390/plants10071284

**Published:** 2021-06-24

**Authors:** Ľuboš Moravčík, Radko Vincúr, Zdenka Rózová

**Affiliations:** 1 Department of Garden and Landscape Architecture, Faculty of Horticulture and Landscape Engineering, Slovak University of Agriculture in Nitra, Tulipánová 7, 949 76 Nitra, Slovakia; lubos.moravcik@uniag.sk; 2 Department of Ecology and Environmental Sciences, Faculty of Natural Sciences, Constantine the Philosopher University in Nitra, Trieda A. Hlinku 1, 949 74 Nitra, Slovakia; zrozova@ukf.sk

**Keywords:** terrestrial laser scanning, finite element model, structural analysis, acoustic tomography, tree breakage prediction, Tilia cordata

## Abstract

This work deals with the innovated complex process of tree risk assessment, from precise geometrical tree shape acquisition to building and analyzing a finite element model under specified load. The acquisition of the 3D geometry of the tree was performed by means of terrestrial laser scanning. Obtained point cloud was optimized and additionally converted to a 3D CAD model, representing the bearing skeleton compound of trunk and main branches. For structural analysis purposes, a finite element model (FEM) was built in the form of beam structure fixed to the ground. Particular beams were defined by geometry, material properties of wood, and cross sections. An acoustic tomography was applied for determination of the precise cross section on investigated locations of an analysis model. Finite element analysis performed on the computational model shows the bearing capacity and deformations of the whole tree structure caused by combinations of load cases like self-weight and static equivalent of wind load. The results of the structural analysis called attention to potentially dangerous places within the tree structure with extreme node displacements or tensile stresses on beams. Thus, we observed a maximal horizontal displacement of 280.4 mm in node N34 and dangerous tensile stress in node N26, where it reaches +23.6 MPa. After filtering some beams with an abnormal cross section geometry, the finite element analysis of the whole tree structure showed the highest tensile stress of +8.8 MPa and highest compressive stress of −8.9 MPa. The suggested method can be applied generally for the prediction of potentially risky tree suspected of breakage and especially for monumental trees, where the presented method can be mostly applicable.

## 1. Introduction

“The cost of prevention is usually much less than the cost of loss and inconvenience of damage” [1].

Trees create pleasant places in built-up urban environments [2]; unfortunately, they can easily be influenced by outer factors. Conifer trees in forest stands are prone to breakage during storm events [3], and sometimes, they fail without previous transmitting of any failure signals. To minimize the potential damage and to avoid a tree’s felling, risk assessment is crucial in managing urban trees [4]. Therefore, trees in urban forests need care provided by arborists. Ideally, arborists would be able to assess combinations of potential load and tree form and estimate the associated probability of failure for the parameters observed [5], but there is a lack of complex methods to make a responsible decision [6].

Since last century, many authors have developed and then also tested their methods, and many producers have manufactured focus-on devices for only one purpose—tree risk estimation. The VTA (visual tree assessment) method published by Mattheck and Breloer [7] is a traditional and frequently used visual assessing method whereby practitioners are able to increase their skills and apply a greater degree of objectivity [8]. In this quantitative evaluation method, they brought the criterion t/R > 0.3–0.35 [9]. A significant stress increase is only expected when hollow occupies more than 70% of stem diameter [7], but according to a reviewing article by Detter et al. [10], above 40% of tested trees with t/R ratio lower than 0.3 were stable.

There was another visual method developed for detecting tree stability—the SIA (static integrated assessment) method [11]. All data are compiled on the basis of device supported SIM (static integrated method), which was collected from more than 4800 pulling tests [10]. Essentially, a modest bending load is applied by means of a cable and winch and the resulting strain is measured through strain gauge [12]. At present, an optical technique wherein small dots are sprayed on the tree surface is often used, especially for numerical analysis. This optical strain measurement method shows the strain distribution of trunks and branches under load [13,14].

During visual types of assessments, it was found that there were significant differences between assessors for the probability of failure and the risk rating [15] and professionals with training and industry credentials also indicated lower risk ratings [16].

Presumption of failure is determined from the bending capacity of the tree, based on geometry and material properties, as well as the anticipated loads and weather [5]. Tree failure can be divided into two main damages: breakage and uprooting [17], where the former can be caused by a stem breakage or branch failure [13]. Resistance assessment against breakage is common at present, but it has reference to limited equipment and unknown input attributes [17]. Failure by uprooting significantly depends on root architecture, which is a complex structure that comprises multiple traits of the root phenotype [18] as well as soil characteristics. Trees can unfortunately be threatened by anthropogenic and natural impacts, where one of the most important factors that decreases the mechanical strength of a tree, and thus increases the risk of a stem collapsing, is decay fungi [4].

The evaluation of trees still has limited ability to directly measure many aspects of tree stability or instability [5]. Non-destructive measurement of the height of plants [19], other dendrometric data, and combining with finite element analysis is possible in a three-dimensional point cloud created from light detection and ranging (LiDAR) scans to obtain non-invasive 3D data of real-world trees at sub-millimeter scale [20,21,22] or by RPAS-based and close-range photogrammetry (CRP), respectively, which can be used to measure precise and detailed dendrometric and phenological parameters [23,24]. Data acquisition with remote sensing is coming to the center of attention, because techniques based on laser and optical sensors provide spatial and metric data. To build a precise geometrical shape and in the case of a very complex geometrical structure such as a tree, using the terrestrial laser scanning (TLS) is the most suitable approach, which is characterized by a high precision over a relatively long distance [25,26]. Among the various kinds of defects that can be assessed visually, decay and cracks are increasingly subjected to detailed tests with a range of devices [8]. There is currently a promising technology being used in the industry—tomography [5]. The most commonly used acoustic tomographs in commercial enterprises are Argus Electronic Picus, Rinntech Arbotom, and Fakopp ArborSonic [27].

The precise tree risk assessment research should be based on biomechanical consideration, which depends on load and stress analysis [28]. Biomechanical models that describe tree movement in wind or predict tree failure can be very useful for practitioners [5]. The correct method to determine the structural resistance of a tree is structural analysis, involving a computational model building, defining material properties of a trunk and branches, as well as a load actuating on the tree structure [26]. These loads and forces affecting trees can be measured and analyzed using static and dynamic methods. Static methods of structural analysis apply a load and then measure the deflection of a beam or part of a structure [14]. More complex models and finite element analyses are being developed with a multimodal approach to represent the dynamics of branches on trees. A review indicates that material properties play only a limited role in tree dynamics and in the form and morphology of the tree and branches, which can influence the dynamics of trees [29]. These numerical strain and stress analyses of tree stem and branches under applied load [13,30] should be confirmed by experimental tests.

The submitted article presents the structural analysis of a single tree, where a complex process was used. Precisely obtained data from a terrestrial laser scanner and acoustic tomograph were transformed in three-step postprocessing and supplemented by numerical parameters to generate a finite element model.

## 2. Results

**Figure 1:**
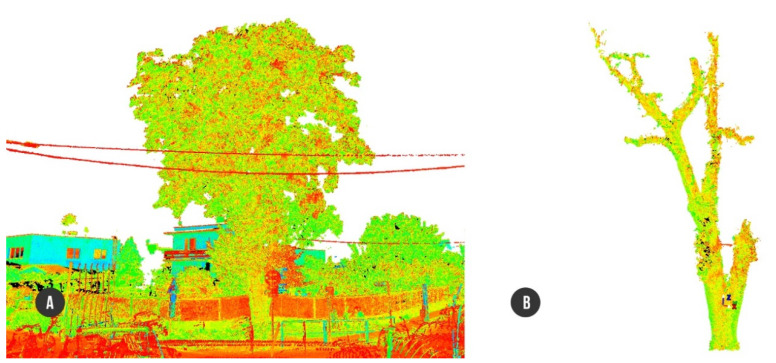
Point cloud model created in HDS Cyclone: (**A**) tree with surroundings; (**B**) separate tree structure ready for conversion to 3D-CAD.

**Figure 2:**
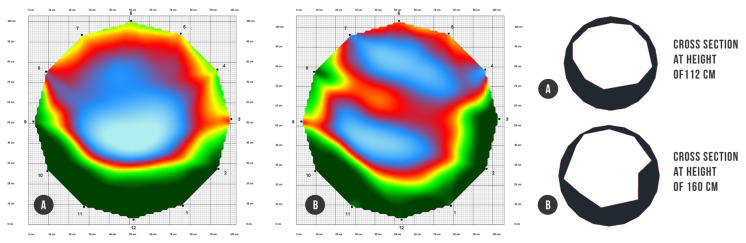
Vectorization of acoustic tomography images of decayed places into closed polylines. Color scheme of tomograms: green (intact wood), red (decayed wood), and blue (cavity or decay): (**A**) cross section at height of 112 cm; (**B**) cross section at height of 160 cm.

**Figure 3:**
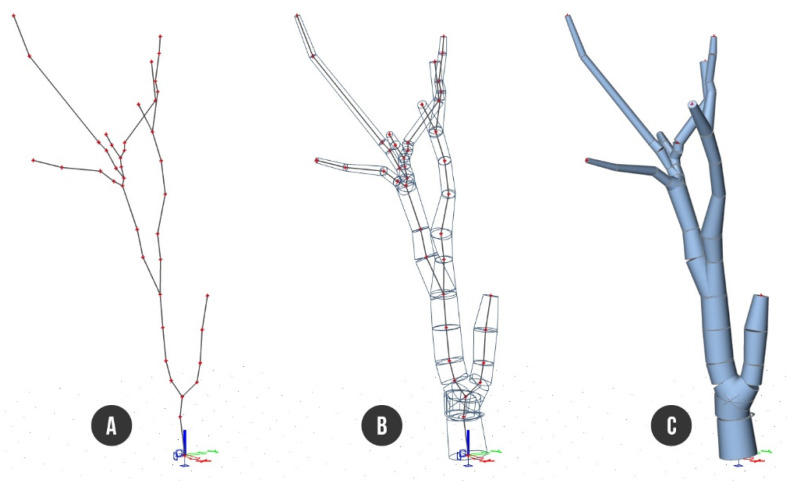
Finite element model of a tree in SCIA 20: (**A**) axial skeleton—beams connected by nodes; (**B**) cross sections of beams—non-prismatic cylinders; (**C**) rendered surface model.

As mentioned in Chapter 4 of this article, we reconstructed the main architecture of both outer and inner body of the single tree *Tilia cordata* Mill. via high precise acquisition hardware of terrestrial laser scanner (outer) (Figure 1) and acoustic tomograph (inner) (Figure 2). Point cloud acquisition of the tree was converted into a 3D CAD model representing an input geometry for subsequent finite element analysis (Figure 3) (FEM file S1).

**Figure 4:**
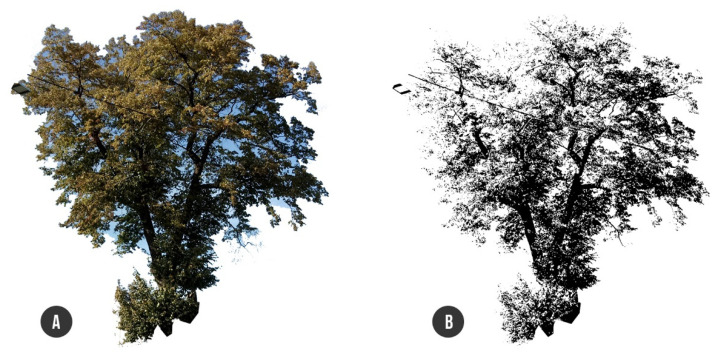
Crown transparency estimation: (**A**) RGB image of the tree crown trimmed by outline; (**B**) RGB image converted to black and white by Phyton script with black to white (transparency) ratio calculation.

**Figure 5:**
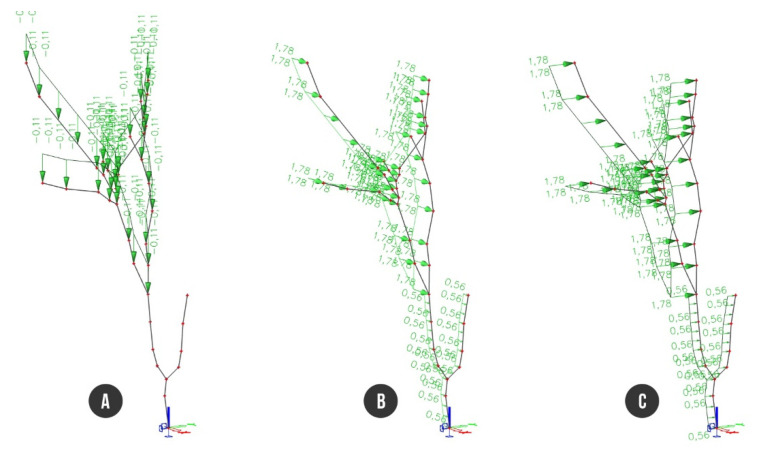
Finite element model of tree with load applied to branches and trunk: (**A**) foliage self-weight of branches; (**B**) static wind load along the X axis; (**C**) static wind load along the Y axis.

The computed rough crown area must be reduced by gaps representing the crown transparency. For that purpose, we used our own program written in Python language aimed at the calculation of the transparent/non-transparent pixels ratio within the crown image (Figure 4) (Text file S1). The reduced non-transparent part of the crown area was computed to 71.14 m^2^ with adequate wind force of 48.42 kN calculated for wind velocity of 33 m/s according to European Standards EN 1991-1-4 [31]. Load cases LC2 (foliage self-weight), LC3, and LC4 (static wind) were proportionally distributed in the form of uniformed load on all beams of the tree structure, i.e., g = 0.11 kNm^−1^ (LC2) and p = 1.78 kNm^−1^ (LC3, LC4), whereas the wind load acting on trunk was considered as p = 0.56 kNm^−1^. The foliage self-weight load was distributed on branches (beams B4–B12) only, excluding the trunk. The load case LC2 had a vertical orientation, while load cases LC3 and LC4 were applied along the horizontal direction of the X or Y axis, respectively. Afterwards, three load combinations were generated from particular load cases. The first combination (C1) represented self-weight of the wood and foliage (LC1 + LC2). The second (C2) and third load combinations (C3) involved whole self-weight with wind load along either the X axis (LC1 + LC2 +LC3) or along the Y axis (LC1 + LC2 + LC4) (Figure 5).

Upon applying the load combination to the beam structure of the tree, the finite element model was completed and prepared for further evaluation of deformations and stresses.

### 2.1. Structural Analysis of Finite Element Model of the Tree

#### 2.1.1. Displacement

The main advantage of the structural investigation into complex finite element models is the possibility to evaluate the deformation of any node and stress at any position of whichever beam of the structure depending on the considered load cases or combinations. In our study, we have evaluated displacements of nodes for load combination C1 (self-weight, no wind), whereby the maximal movement of 25.3 mm (N34) was calculated. Load combination C2 and C3 (wind load involved) caused much bigger horizontal displacement, namely 280.4 mm (wind along the X direction) and 265.8 mm (wind along the Y direction), respectively.

#### 2.1.2. Stress

**Figure 6:**
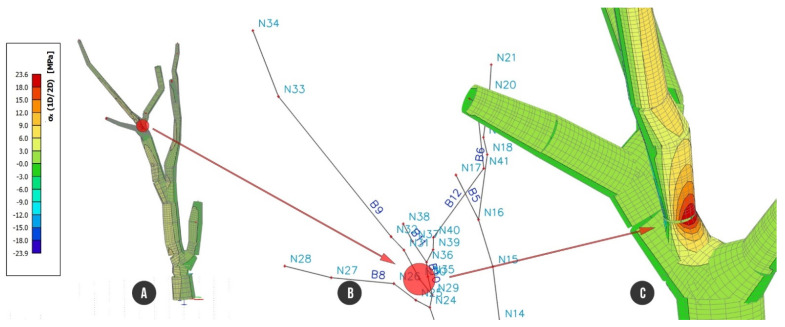
The most stressed place of the tree resisting against self-weight and wind load, which was filtered out from the results due to abnormality of the cross section: (**A**) whole tree structure; (**B**) axial beam structure focused on node N26 with the biggest tensile stress; (**C**) detailed view of node N26 with colored representation of stress, max. value of stress of 23.6 MPa.

Analyzing the beam’s strain caused by self-weight including foliage (C1) recovered one position within the finite element model of the tree structure with bigger stress than the rest of the tree. Node N26 located near the branching point on the branch (B9) showed stress up to 3.1 MPa, evaluated without wind load. Values of stress in the tree beam structure during the hurricane wind (33 ms^−1^) showed the same dangerous place. Wind load along the X axis (C2) resulted in up to 22.9 MPa stress on the tensile branch edge, and for wind load along the Y axis, the stress reached a maximum of 23.6 MPa (Figure 6).

The stress close to node N26 reached up to +23.6 MPa (Figure 6) where the beam had a significantly lower diameter of cross section than surrounding parts of beam. Even though the 3D geometry of reconstructed model is correct according to performed visual check, we filtered data for the stress analysis. In accordance with this geometrical abnormality, we slightly modified the model by splitting the beam B9 into two parts. The problematic beam was excluded from presentation of stress development and thus we observed a smaller stress range on the whole beam structure.

**Figure 7:**
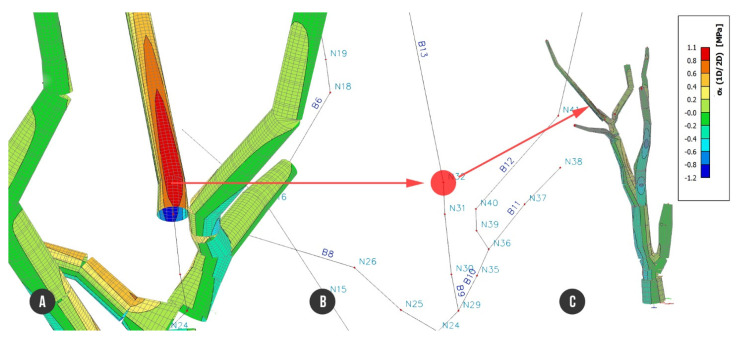
The most stressed place of the tree resisting against load combination C1 (self-weight + foliage): (**A**) detailed beam structure with colored representation of stress, max. positive value of stress of +1.1 MPa; (**B**) axial beam structure focused on node N32 with the biggest tensile stress; (**C**) entire tree model.

Finite element analysis of load combination C1 showed low tensile stress up to +1.1 MPa and compression stress up to −1.2 MPa. On the trunk part of the tree along beam B1 and B3, we can observe the compression stress ranging from around −0.4 MPa to −0.6 MPa and a beam on the bottom side of decayed cross section with the stress from −0.6 MPa to −0.8 MPa. The biggest tensile stress of +1.1 MPa was reached on beam B13 and the compression stress of −1.2 MPa on the opposite side of the beam (Figure 7).

**Figure 8:**
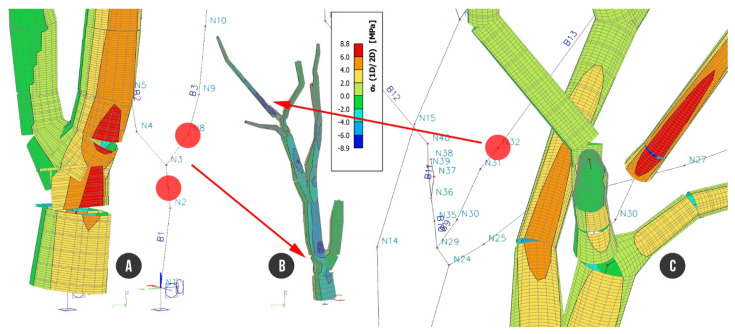
Tree structure with analyzed stress under load combination C2: (**A**) detailed view of trunk; (**B**) entire tree structure; (**C**) node N32 with the highest tensile stress of +8.8 MPa.

Load combination C2 defined by self-weight including foliage and wind along the X direction showed stresses up to +8.8 MPa and −8.9 MPa, respectively. A comparable value of stress was reached on more beams of the tree structure. Tensile stress of +8.8 MPa runs through B1 and continues to beam B3 through nodes N8 and N9, where the stress is decreased back to +4 MPa up to +6 MPa. Tensile stress of +8.8 MPa was recorded on branch B7, at node N23. Evaluating beam B13 under load combination C2 showed tensile stress reaching up to +8.8 MPa and compression stress up to −8.9 MPa on the opposite side of the branch. Compression stress of −8.9 MPa was also recorded at node N2 (B1), where decayed hollow of trunk cross section started, and then at node N8 (B3), above the location where the trunk was divided into two parts (Figure 8).

**Figure 9:**
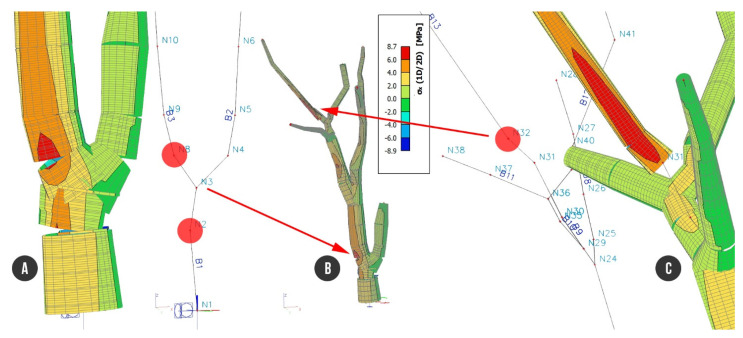
Maximal tensile stress reaching +8.7 MPa caused by load combination C3: (**A**) positions of nodes N1 and N8 on the trunk; (**B**) entire tree structure; (**C**) detailed view of highest branch of the tree with value of tensile of +8.7 MPa.

The last load combination C3, where the stress is caused by self-weight, foliage, and wind along the Y direction, showed the maximum tensile stress of +8.7 MPa and compressive stress of −8.9 MPa. This maximum stress was reached on the trunk beam, in particular at node N2 (B1), with detected decayed hollow, and as well as at node N8 (B3). The maximum compressive stress of −8.9 MPa was also recorded on the opposite sides of the trunk. Smaller stress values in a range of +4 MPa to +6 MPa were analyzed around node N23 (B7) and nodes N12 and N14 belonging to beam B4. The highest branch of the tree (B13) showed tensile stress up to +8.7 MPa and compressive stress up to −8.9 MPa, and then it decreased to the range of +4 MPa to +6 MPa upwards to node N33 and downwards to node N31 (Figure 9).

## 3. Discussion

The submitted study presents a complex and more accurate approach to evaluate both strain and stress behavior of the analyzed tree and call arborists’ attention to the weakest part of the tree structure. Our results showed exact displacements and stresses consequent upon three combinations of applied load cases. After exempting node N26 from evaluation (with an abnormal cross section diameter), where the stress reached up to +23.6 MPa, the structural analysis showed a maximal tensile stress up to +8.8 MPa. In the literary overview, we can find compressive strength for *Tilia cordata* set up to 20 MPa [11] or similar species *Tilia americana* up to 15.3 MPa [32] and *Tilia vulgaris* with the value of 26.1 MPa [33]. Based on values resulting from the provided structural analysis, we cannot predict significant breakage probability, not even of the highest tree branch under strong wind pressure.

The authors of [13] provided a comparison between numerical analysis and force applied on the four branches from four trees *Tilia cordata* L. Branches were chosen with different geometrical parameters to verify FE simulations. They differ in diameter, shape of cross section, tapering, and angle of attachment. Parametrical numerical simulations of branch mechanical response to loading were carried out using finite element software ANSYS, where segments of the branches were built from tapered beam elements. Several cross sections were made manually, not using a precise acquisition device, like TLS. They used the same G_LT_/E_L_ ratio for the estimation of shear modulus, as mentioned in our method. The numerical model was verified by the experimental assessment of branch bending. The maximum deflection ranged from 12.6 mm to 33.7 mm depending on the branch. The pulling experiment showed that there can be a difference in the branch’s deflections between the simulation and the test in the range of 0.5% to 26% and may be explained by the variable modulus of elasticity of branches.

Another numerical analysis [30] was provided on the tree species *Aesculus hippocastanum* L., where authors measured data for validation of the FE model regarding tree response. The standard pulling test using elasto-inclino measurement was supplemented by optical measurements. The parametric linear static structural model of the tree mechanical response was built using FE software ANSYS and the 3D model had simplified stem and root-plate geometry divided into 192 segments in total, which can predict high accuracy of the geometrical model. Simulated data were compared with data from mechanical sensors and marker tracking, which showed the difference between experimental devices and the FE model results. Symmetrical response of the model did not fit the measurements. They found relative errors between the model and measurement in the range from −14% to −33%, with an average of 22% and variation of 22%. As in our case, they performed filtering of data. The points closest to the tree base were excluded from the validation because they did not provide representative results.

The combination of terrestrial laser scanning and finite element analysis was presented in the work of [22], where a comparison of field data collection and model from TLS was provided. They measured the bending strains produced on 21 trees. They manually extracted trees from point cloud and made cylinders by QSM algorithm application. The amount of cylinders was reduced without significant affecting the total woody volume of the tree. The finite element model was built in Abaqus and each cylinder was represented as a two node Euler–Bernoulli beam element. They applied the dynamic simulation of the tree’s response to wind forcing. It is very useful to measure trees in the winter season, because of an absence of leaves, which makes the modelling simpler. All modelled trees displayed some peaks that were not evident in the field data. Amount of simplification can increase the degree of deviation. The simplification step used to reduce the number of cylinders had a large effect on the result.

These articles deal with comparisons of computed analysis against the data measured in the field. The range of deviation can suggest that data analyzed from the finite element model should be confirmed with data obtained from real world trees. It is more than probable in our case that we will find some new locations on the tree during the real test, and this will help us set-up our FE model to avoid bigger deviations.

For further research, it is necessary to develop a computerized or more user friendly semi-automated conversion of tree point cloud to a 3D-CAD model, which can significantly improve the overall workflow. Additionally, a bigger effort should be dedicated to retrieving more relevant data of the mechanical properties of green wood, especially with the constant moisture content of about 50%, and implementing them in the evaluation process. The last, but not least, goal is to experimentally confirm data obtained from finite element analysis by comparison with tree pulling tests, as well as to find out the range of deviation and possibilities to increase the finite element model accuracy.

Owing to the huge amount of impact factors and complexity of tree architecture, the performed analysis focused on tree risk assessment will be subject of comprehensive research in the future.

## 4. Material and Methods

### 4.1. Equipment

The main goal of our study is the determination of mechanical stress influencing a single tree, as well as to find the breaking point of the weakest body part of a tree, of trunk and branches particularly. Precise data of outer 3D-shape of tree are needed for the definition of accurate tree geometry. Therefore, the acquisition method by means of terrestrial laser scanning (TLS) was used. TLS is considered to be the most accurate method for 3D reconstruction of complex objects, and is especially used in the building industry. We worked with the currently used Leica ScanStation P20, high speed pulse laser scanner, which includes modern waveform digitizing (WFD) technology. It is capable of capturing a long range distance up to 120 m and, furthermore, is able to scan up to 1 million points per second with the high acquisition accuracy. The scanner has the ability to rotate in 360° angle horizontally and move in the range of −55° to +90° vertically, which ensures a point cloud with an almost panoramic environment around its axis. The amount of captured points depends on resolution settings, moving up to 0.8 mm at 10 m. With increasing resolution, the time of scan acquisition increases from 50 s to 16 min, respectively. Targets on tripods may be used for better accuracy when registering scans into a complex one.

In an effort to obtain the most precise possible risk analysis for the breakage of the focused tree, data received from the outer 3D geometry are not enough, especially when the tree reports visible or obvious injuries inside the structure. This issue should be taken into account with an emphasis on trees in urban environments. In these situations, an acoustic tomograph, frequently exploited by woodworkers, is used. Our study worked with one of the common devices Fakopp ArborSonic 3D. It is based on spreading sound across a wood structure. The slower a sound spreads through the wood structure, the higher the possibility of defects. Sensors can send and receive signals from each other, which allows to detect the sizes and locations of a hollow or a decay. Affiliate software processes measured speeds and represented them by color sensitivity in the range of green for healthy wood to blue color for detected hollow.

### 4.2. Point of Interest

Small-leaved lime tree (*Tilia cordata* Mill.) was chosen for modeling and investigation within this study. The selected mature tree is located in the village Lužianky, Nitra, Slovakia (GPS 48.330030, 18.037178) right next to the intersection of two busy roads. As a single tree, it represents the tallest object in close surroundings, so it is exposed to external weather conditions, especially wind.

### 4.3. Methods

#### 4.3.1. Data Acquisition

The submitted study introduces an integrated workflow process from geometric acquisition of the single tree to finite element analysis. This brings a new method into the field of tree care and risk assessment. Because of the unknown root structure and soil characteristics, the method is focused on above ground tree parts’ breakage influenced by self-weight and static wind. The process of modelling and structural analysis of a single tree was carried out in three main steps: point cloud acquisition and processing supplied by the TLS, conversion of point model into 3D CAD model consisting of the bearing axial skeleton and cross-section definition (trunk and branches), and the structural analysis of the finite element model with respect to acting load.

Data collected from TLS were worked out on 31 July 2020, although leafless crown scanning is recommended to improve the geometric accuracy of the tree skeleton. After estimating the site situation, we decided to measure the selected tree from six different scan positions around the crown, at a variable distance of 2 m to 12 m from the trunk, to capture the significant shapes of trunk and branches. Every Scanstation position was set up in the default field of view setting, 360° horizontally and 290° (−55° to +90°) vertically. The scanning resolution and density of point cloud were adjusted to 6.3 mm@10 m. Point cloud acquisition lasted 1 min 49 s for each scan.

#### 4.3.2. Data Postprocessing

Terrestrial laser scanning data represented by raw point clouds of points were transferred into the workstation with HDS Cyclone (version 9.4, developed by Leica) software to provide a fusion of all six scans into a complex one by the means of a visual registration procedures. Every point from the point cloud environment carries information about the XYZ coordinates and the intensity of reflected light. After scans aligning with the final 23 mm deviation (vector error) caused by manual merger, we performed a basic cleaning from digital noises caused by surrounding environment around the tree. Whole cloud size of 180,914,896 points was manually reduced to 16,921,877 points, representing the main tree architecture (Figure 1). Afterwards, the attention was concentrated on CAD model preparation.

#### 4.3.3. Point Cloud to CAD Conversion

There are several ways to transform the tree point cloud to a 3D-CAD model. Often, used manual conversion is a time-consuming option; however, keeping the process under control is an important advantage. Some authors developed so-called semi-automatic techniques for point cloud rendering into CAD supported software. A lot of them, based on different algorithms and programming languages, are not user friendly for inexperienced users. We tried to apply our data to two similar QSM (quantitative structure model) applications: simple forest [34] and 3D forest [35]. More point cloud models of different trees were tested. A successful result came in the case of a continuous trunk-shaped young tree *Paulownia tomentosa* from a research plantation, where the biomass values were discovered. Unfortunately, the result of automated segmentation for the adult tree *Tilia cordata* with a very random curved shape of trunk and branches was not inconvenient enough.

Therefore, the three-dimensional model was manually divided by 44 representative cross sections from the bottom of the trunk to the highest skeleton branches, and then redrawn through 3D polylines. As we needed to trace the tree’s architecture, we focused on the generation of cross sections in the point of the model, where the shapes of stem or branches, eventually, were curved.

#### 4.3.4. Computational Model of the Tree

The investigated tree was analyzed within the SCIA Engineer (Version 20, developed by Nemetschek) program based on the finite element method (FEM). From an FEM point of view, the computational model of the tree is a space beam structure fully fixed to the ground with the elimination of all degrees of freedom (three translations and three rotations related to the XYZ axis) in the supporting point. The final finite element model of the tree structure consists of 13 beams connected by 42 nodes.

SCIA Engineer allows the user to define a 1D member (beam) whose cross section varies arbitrarily along the 1D member length. 1D member of arbitrarily variable cross section can be divided into segments called spans. Each span has specific properties that are absolutely independent of the properties of adjacent spans. The cross section of the span and its change can be defined in several ways; however, in our case, we used the two cross sections method. Two cross sections corresponding to the two end-points of the span are defined. The cross section varies over the span from one section to the other, reflecting the geometrical properties of tree skeleton as precisely as possible.

Particular beams were defined through 44 different cross sections and divided into trunk and branches parts, thence three beams (B1–B3) represented the trunk and the remaining 10 beams (B4–B13) formed the structure of branches. Measured circular cross sections were attached to particular beams in the connecting nodes building tapered non prismatic cylinders (Figure 3).

#### 4.3.5. Wood Material Attributes

For properly working FEM analysis, the material properties of wood have to be added correctly. Important attributes influencing the behavior of the tree under structural analysis as a modulus of elasticity, modulus of rigidity, and wood density were defined. It is easy to find a database of timber properties for any species; however, there is weaker documentation of green wood, meaning the mechanical properties of wood by at least 50% moisture content; therefore, we decided to use the closest values available to our small-leaved lime tree. The wood density value of greenwood by 50% moisture content was adapted from Niklas and Spatz [33] for *Tilia vulgaris* and set to 657 kg/m^3^. Modulus of elasticity (E) was taken from the same table database [33]. Owing to a lack of input data, we chose a similar species *Tilia vulgaris* known as a hybrid of both *Tilia cordata* and *Tilia platyphyllos*. The modulus of rigidity was derived from E_L_ by the ratio G_LT_/E_L_= 0.046, featured in Kretschmann [32]. Because of the unavailability of the green wood ratio or value for modulus of rigidity, we had to use the value for basswood with 12% moisture content.

#### 4.3.6. Tree Geometry

The basic geometry of the tree was imported into the structural analysis environment in the form of a DXF (data exchange file) representing the tree skeleton geometry consisting of the trunk and branches, which mostly participate in the bearing capacity of the tree. The basic skeleton was prepared from a point model within the postprocessing of primary point clouds obtained from a 3D Leica P20 Scanstation scanner. When building a representative axial skeleton of the tree, it was important to divide a whole structure into a suitable number of straight elements of different length according to the heading of the growth units.

#### 4.3.7. Determining of the Precise Cross Section of Beams

The outer shape of the cross section in the specified place of the tree skeleton was obtained from the point cloud model, just using the “quick slice“ function (Cyclone software) and creating the representative closed outline polygon with an orientation perpendicular to the longitudinal beam axis. In the suspicious places where an internal damage of wood was expected, precise beam cross sections were determined by means of ArborSonic 3D acoustic tomograph (Figure 2). The majority of beams (41) were recognized as undamaged with full circle cross sections. Only the beam between nodes N2 and N3 had decayed hollow cross sections applied, whereby the images of particular cross sections were generated representing real intact, decayed, and hollow parts of timber. Exported bitmap forms of cross sections were additionally converted to DXF vector files to prepare suitable input data for finite element analysis (Figure 3) in the form of vectorized closed polylines. The whole beam structure of the tree was finally defined by 44 different cross sections.

#### 4.3.8. Application of Load Cases (LCs)

The load acting on the whole tree structure consists in principle of two types: self-weight and climate load. In our study, we applied three load cases (LCs): self-weight of wood (LC1), self-weight of the foliage (LC2), and wind load (LC3, LC4). The first load case is fully automatically generated by the SCIA software from the bulk density of wood. For foliage self-weight (LC2) determination, an appropriate allometric equation [36] was applied, resulting in a total crown foliage weight of 301.61 kg. Wind load was calculated according to Eurocode [31], whereby the entire crown area exposed to wind pressure Aw = 100.2 m^2^ was taken into account and calculated from the closed polyline representing the vectorized crown outline reduced by the transparency rate.

## 5. Conclusions

The main point of the presented method should be to provide exact measurements in longer time periods, especially for assessment of the conditions of monumental trees. Tree static assessment or estimation of tree failure probability is still an unsolved issue. There are many visual assessment methods as well as device supported methods to measure and assess the tree stability on the location; however, they often come with a high range of simplification and focus on the specific spot on the tree. Our methodology brings a new vision to this topic with the use of highly precise up-to-date technology provided by terrestrial laser scanning and acoustic tomography. Obtained data compose the main building parts for finite element analyses via Scia Engineer software, whereby other FE software (ANSYS, Abaqus, and so on) is available as well.

Based on the obtained analysis results, we found several tree locations with tensile stress reaching +8.8 MPa when load combination C2 was analyzed. In consideration of the literature [11,32,33], it does not represent a risk of breakage failure; however, this is a hypothesis only, because we should confirm our computational data by real tests.

## Supplementary Materials

The following are available online at https://www.mdpi.com/article/10.3390/plants10071284/s1, FEM file S1: FEM_tilia_updated_v2.esad, Text file S1: Ttransparency_Phyton_script.

## References

[1] Cox D. (2009). Tree Risk Assessment and Hazard Abatement.

[2] Terho M., Hallaksela A.M. (2005). Potential hazard characteristics of *Tilia*, *Betula*, and *Acer* trees removed in the Helsinki City Area during 2001–2003. Urban For. Urban Green..

[3] Rinn F. (2013). Shell-wall thickness and breaking safety of mature trees. West. Arborist.

[4] Heikura T., Terho M., Perttunen J., Sievänen R. (2008). A computer-based tool to link decay information to 3D architecture of urban trees. Urban For. Urban Green..

[5] Dahle G., Grabosky J., Kane B., Miesbauer J., Peterson W., Telewski F.W., Koeser A., Watson G.W. (2014). Tree Biomechanics: A White Paper from the 2012 International Meeting and Research Summit at the Morton Arboretum (Lisle, Illinois, U.S.). Arboric. Urban For..

[6] Bond J. (2010). Field tree load analysis: A basic method for arborists. West. Arborist.

[7] Mattheck C., Breloer H. (2003). The Body Language of Trees. A Handbook for Failure Analysis.

[8] Lonsdale D. (2003). Overview of techniques and procedures for assessing the probability of tree failure. Tree Statics Tree Dyn..

[9] Bond J. (2006). Foundations of Tree Risk Analysis: Use of the t/R ratio to Evaluate Trunk Failure Potential. Arborist News.

[10] Detter A., Brudi E., Bischoff F. (2005). Statics Integrated Methods. Results from Pulling Tests in the Past Decades. La Vision del Árbol Urbano. Actas del 9° Congresso de Arboricultura, Barcelona, AEA, Spain.

[11] Wessolly L., Erb M. (2016). Manual of Tree Statics and Tree Inspection.

[12] Sinn G., Wessolly L. (1989). A Contribution to the Proper Assessment of the Strength and Stability of Trees. Arboric. J..

[13] Vojáčková B., Tippner J., Horáček P., Praus L., Sebera V., Brabec M. (2019). Numerical Analysis of Branch Mechanical Response to Loading. Arboric. Urban For..

[14] James Kenneth R., Moore John R., Slater D., Dahle G.A. (2017). Tree biomechanics. Cab Rev..

[15] Coelho-Duarte A.P., Daniluk-Mosquera G., Gravina V., Vallejos-Barra Ó., Ponce-Donoso M. (2021). Tree Risk Assessment: Component analysis of six visual methods applied in an urban park, Montevideo, Uruguay. Urban For. Urban Green..

[16] Koeser Andrew K., Smiley E. (2017). Thomas. Impact of assessor on tree risk assessment ratings and prescribed mitigation measures. Urban For. Urban Green..

[17] Luděk P. (2006). Plošné Poškození Lesů Způsobené Povětrnostními Vlivy.

[18] Colchado-López J., Cervantes R.C., Rosas U. (2019). A Linear Model to Describe Branching and Allometry in Root Architecture. Plants.

[19] Wang Y., Chen Y. (2020). Non-Destructive Measurement of Three-Dimensional Plants Based on Point Cloud. Plants.

[20] Hu S., Li Z., Zhang Z., He D., Wimmer M. (2017). Efficient tree modeling from airborne LiDAR point clouds. Comput. Graph..

[21] Paulus S., Schumann H., Kuhlmann H., Léon J. (2014). High-precision laser scanning system for capturing 3D plant architecture and analysing growth of cereal plants. Biosyst. Eng..

[22] Jackson T., Shenkin A., Wellpott A., Calders K., Origo N., Disney M., Burt A., Raumonen P., Gardiner B., Herold M. (2019). Finite element analysis of trees in the wind based on terrestrial laser scanning data. Agric. For. Meteorol..

[23] De Petris S., Sarvia F., Borgogno-Mondino E. (2020). RPAS-based photogrammetry to support tree stability assessment: Longing for precision arboriculture. Urban For. Urban Green..

[24] Mokroš M., Výbošťok J., Tomaštík J., Grznárová A., Valent P., Slavík M., Merganič J. (2018). High Precision Individual Tree Diameter and Perimeter Estimation from Close-Range Photogrammetry. Forests.

[25] Moravčík Ľ. (2014). Use of terrestrial laser scanning in the precise determination of geometrical features changes of Ginkgo biloba L. Plants in Urban Areas and Landscape 2014.

[26] Ľuboš M. (2018). Precise digital model and finite element analysis of a single tree. Plants in Urban Areas and Landscape 2018, Hokovce.

[27] Cristini V., Tippner J., Vojáčková B., Paulić V. (2021). Comparison of Variability in Results of Acoustic Tomographs in Pedunculate Oak (*Quercus robur* L.). BioResources.

[28] Nelda M., Jim C. (2009). Tree Risk Assessment. What We Know (and What We Don’t Know). Arborist News.

[29] James K.R., Dahle G.A., Grabosky J., Kane B., Detter A. (2014). Tree Biomechanics Literature Review: Dynamics. Arboric. Urban For..

[30] Vojáčková B., Tippner J., Horáček P., Sebera V., Praus L., Mařík R., Brabec M. (2021). The effect of stem and root-plate defects on the tree response during static loading-Numerical analysis. Urban For. Urban Green..

[31] EN 1991-1-4. Eurocode 1: Actions on Structures. Part 1–4: General Actions. Wind Actions. [The European Union Per Regulation 305/2011, Directive 98/34/EC, Directive 2004/EC]. https://www.phd.eng.br/wp-content/uploads/2015/12/en.1991.1.4.2005.pdf.

[32] Kretschmann David E. (2010). Mechanical Properties of Wood. Wood Handbook. Wood as an Engineering Material.

[33] Niklas K.J., Spatz H.C. (2010). Worldwide Correlations of Mechanical Properties and Green Wood Density. Am. J. Bot..

[34] Hackenberg J. (2019). The Simple Forest Handbook. A User Guide for QSM Building.

[35] Petrov M., Krůček M., Trochta J., Král K. (2020). 3D Forest User Guide.

[36] McPherson E.G., van Doorn N.S., Peper P.J. (2016). Urban Tree Database and Allometric Equations.

