# Supplementary material for: Analysis of the Static Behavior of a Single Tree on a Finite Element Model
Source: Plants (Basel). 2021 Jun 24;10(7):1284. doi: 10.3390/plants10071284 (PMC8309158; doi:10.3390/plants10071284)
Supplement: Supplementary file 1 [file plants-10-01284-s001.zip › Ttransparency_Phyton_script.rtf]

"""
Created on Sat Jan  2 19:33:34 2021
@author: LM+RV
"""

from PIL import Image
#import cv2

img = Image.open('Tree_transp.png')
datas = img.getdata()

black_white = 0
transparent = 0

newData = []
for item in datas:
    if  item[3] == 0:
        transparent += 1
    else:
        black_white += 1

print('transparent=' + str(transparent) + '  black_white=' + str(black_white))
print('transparent + black_white =' + str(transparent + black_white))
print()

col = Image.open(' Tree_transp.png')
gray = col.convert('L')

treshold = 100 # setup the treshold value

bw = gray.point(lambda x: 0 if x<treshold else 255, '1')
bw.save(' Tree_transp_T_bw.png')

img = Image.open('Tree_transp_T_bw.png')

# Convert Image into RGB 
img = img.convert('RGB') 

black = 0
white = 0

for pixel in img.getdata():
    if pixel == (0, 0, 0): # if your image is RGB (if RGBA, (0, 0, 0, 255) or so
        black += 1
    else:
        white += 1
        
white1 = white - transparent
ratio_b_to_w1 = (black/(black + white1))

print("whole picture:") 
print('black=' + str(black)+', white='+str(white))
print('SUM_of_black+white = ' + str(black + white))
print()
print("picture without transparent pixels:") 
print('black=' + str(black)+', white1='+str(white1))
print('SUM_of_black1+white1 = ' + str(black + white1))
print()
print('treshold = ' + str(treshold))
print('ratio_b1_to_w  = ' + str(ratio_b_to_w1))
